# A gain-of-function CCR4 mutations in adult T-cell leukemia/lymphoma (ATL) enhance the chemotactic abilities and P13K/AKT activation

**DOI:** 10.1186/1742-4690-12-S1-O31

**Published:** 2015-08-28

**Authors:** Masao Nakagawa, Roland Schmitz, Wenming Xiao, Yandan Yang, Thomas A Waldmann, Louis M Staudt

**Affiliations:** 1 LYMB, CCR, NCI, Bethesda, MD, USA

## 

High expression of the CC chemokine receptor 4 (CCR4) has been identified as a hallmark gene in ATL-an aggressive peripheral T-cell neoplasm. CCR4 is a chemokine receptor, which has a critical role in immune cell trafficking including Th2, T-regs and skin homing memory T-cells. CCR4 ligands, CCL17 and CCL22 are produced in lymph nodes and skin from dendritic cells, macrophages and Langerhans cells. Most ATL cases express surface CCR4 (90%) and infiltrate lymph nodes and skin. We performed RNA-Seq on two primary ATL cases and discovered recurrent nonsense mutations in CCR4. Through an extended analysis using Sanger sequencing, CCR4 mutations were detected in 14/53 ATL samples (26%) and consisted exclusively of nonsense or frameshift mutations that truncated the coding region at C329, Q330 or Y331 in the carboxyl-terminus. We hypothesized that the mutant CCR4 isoforms might enhance chemotaxis of the affected cells to CCR4 ligands. A chemotaxis assay using 32D beta, a mouse myeloid cell line and ED40515 (+), an ATL cell line, clarified that the ectopic expression of CCR4-Q330* but not CCR4-WT transduced cells enhanced the chemotactic ability of the transduced cells toward CCL17 and CCL22. To define the mechanism of the enhanced chemotactic ability, we examined the change in surface CCR4 levels after CCL22 exposure in CCR4-WT-and CCR4-Q330*-reconstituted ED40515 (+) cells. Compared with CCR4-WT, CCR4 internalization in CCR4-Q330*-reconstituted cells was significantly impaired. Thus, the ATL CCR4 mutants manifested impaired desensitization by ligand, which likely contributed to the enhanced chemotaxis of cells bearing these mutants. We explored the influence of the ATL CCR4 mutants on PI(3) kinase (PI3K)-dependent activation of AKT since it has been reported that binding of CCL22 to CCR4 activates AKT in CEM leukemic T-cells and in human Th2 cells. CCR4-Q330* reconstituted ED40414 (+) cells showed strong activation of AKT with CCL22 ligation compared with CCR4-WT reconstituted cells. The AKT activation was inhibited by the pan-P13K inhibitor, BKM120, indicating that the CCR4-mediated AKT activation was PI3K dependent. Lastly, we tested whether the acquisition of CCR4 mutations by ATL cells imparts a selective growth advantage relative to cells with wild-type CCR4. CCR4-Q330*-reconstituted cells had a selective growth advantage in the presence of CCL22, supporting, at least in part, the hypothesis that CCR4 mutations provided the affected cells with a positive selection pressure through CCL22 ligation that contributed to the ATL pathogenesis. Our findings provide a rationale for a clinical trial to test whether inhibition of CCR4 signaling might have a therapeutic potential for patients with ATL.

